# Association between perfluoroalkyl substances concentration and bone mineral density in the US adolescents aged 12-19 years in NHANES 2005-2010

**DOI:** 10.3389/fendo.2022.980608

**Published:** 2022-10-05

**Authors:** Xianmei Xiong, Baihang Chen, Zhongqing Wang, Liqiong Ma, Shijie Li, Yijia Gao

**Affiliations:** ^1^ The First Clinical School of Guangzhou University of Chinese Medicine, Guangzhou, China; ^2^ The First Affiliated Hospital, Guangzhou University of Chinese Medicine, Guangzhou, China

**Keywords:** perfluoroalkyl substances, perfluorooctanoic acid, perfluorooctane sulfonic acid, perfluorohexane sulfonic acid, perfluorononanoic acid, bone mineral density

## Abstract

**Background:**

Reports on the association of perfluoroalkyl substances (PFASs) exposure with adolescent bone health are scarce, and studies have primarily targeted maternal serum.

**Objective:**

We evaluated the relationship between autologous serum perfluorooctanoic acid (PFOA), perfluorooctane sulfonic acid (PFOS), perfluorohexane sulfonic acid (PFHxS) and perfluorononanoic acid (PFNA) levels and bone mineral density (BMD) in adolescents.

**Methods:**

We analyzed data from 1228 adolescents aged 12-19 years in the National Health and Nutrition Examination Survey (NHANES) 2005-2010 and used multiple regression analysis to identify the relationship between serum PFOA, PFOS, PFHxS, and PFNA concentrations and total femur, femoral neck, and lumbar spine BMD, in addition to multiple stratified subgroup analyses.

**Results:**

The mean age of participants was 15 years, males had higher serum PFAS concentrations than females. The results of multiple regression analysis showed that the natural log(ln)-transformed serum PFOA, PFOS, and PFNA concentrations were negatively correlated with total femur, femoral neck, and lumbar spine BMD (all p < 0.05), and ln-PFHxS was positively correlated with total femur and femoral neck BMD (all p< 0.05). In males, ln-PFOA was negatively associated with total femur and lumbar spine BMD (all p< 0.05), ln-PFOS was associated with the reduced total femur, femoral neck, and lumbar spine BMD (all p< 0.05), while ln-PFHxS and ln-PFNA were not observed to correlate with BMD at these three sites. In females, both ln-PFOA and ln-PFOS were negatively correlated with total femur and lumbar spine BMD (all p< 0.05), ln-PFHxS is associated with the increased total femur and femoral neck BMD (all p< 0.05), and ln-PFNA was negatively correlated with total femur and femoral neck BMD (all p< 0.05), most of the associations were confined to females. The associations of ln-PFOS with femoral neck BMD and ln-PFNA with total femur BMD were more significant in those who were overweight/obese and had anemia, respectively (all p for interaction < 0.05).

**Conclusions:**

In this representative sample of US adolescents aged 12-19 years, certain PFAS were associated with lower bone mineral density, and most of the associations were confined to females. The negative effect of PFAS on BMD is more pronounced in those who are overweight/obese and have anemia. However, further studies are needed to confirm this finding.

## Introduction

Osteoporosis is one of the most common skeletal diseases and a very important public health problem in populations worldwide, characterized mainly by low bone mineral density, which predisposes to fractures in the affected skeletal areas (1). The critical period of skeletal development during adolescence is important for lifelong bone health because bone mass increases rapidly during adolescence and peaks in late adolescence (2, 3), and peak bone mass during this period may have a significant impact on the onset and diagnosis of osteoporosis in later life (4).

Perfluoroalkyl substances (PFASs) are one of the most stable classes of chemicals in industrial history and have become widespread persistent environmental pollutants since the 1950s due to their widespread use and presence in items we use every day, as well as their long-term and stable presence in the environment (5). PFAS can be exposed to humans and accumulate in the body through a variety of pathways (6, 7), and have been reported to be able to be detected in 95% of the American population (8). Perfluorooctanoic acid (PFOA), perfluorooctane sulfonic acid (PFOS), perfluorohexane sulfonic acid (PFHxS), and perfluorononanoic acid (PFNA), the most commonly used PFASs, have been studied extensively. Although the use PFASs is now widely restricted (www.oecd.org/officialdocuments), a significant percentage of the global population is still exposed to them. PFAS have been classified as endocrine-disrupting chemicals (EDCs) (9), which together with other EDCs have been shown to be strongly associated with a wide range of human health issues including male and female reproductive health, obesity and metabolism, neurodevelopment, and bone health (10, 11), however, few studies have reported on the effects of such environmental pollutant exposures on adolescent bone health (12, 13), and previous studies on PFAS exposure in adolescent bone health have only primarily collected maternal prenatal PFAS exposure levels (14, 15), therefore the extent of the effect of different PFAS on BMD in adolescents is unclear. However, on the basis of the limited available data suggesting a negative association between PFAS exposure and BMD, we proceeded to test the hypothesis that higher PFAS concentrations are associated with lower BMD in the NHANES 2005-2010 cross-sectional survey of adolescents aged 12-19 years.

## Methods

### Study methods and participants

**Figure 1:**
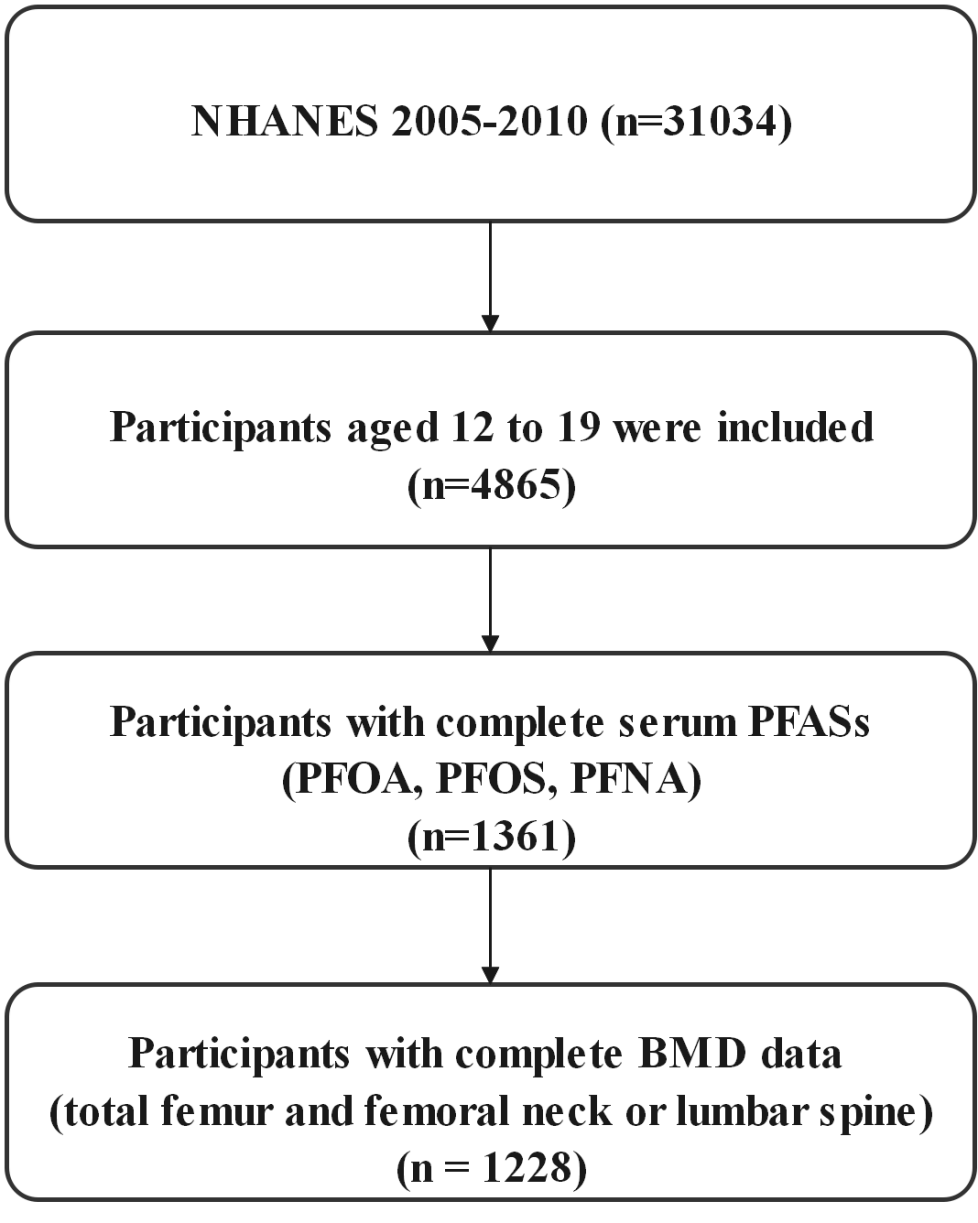
Flow chart algorithm.

NHANES is a nationally representative cross-sectional survey of the health and nutritional status of civilians, noninstitutional adults, and children conducted by the Centers for Disease Control and Prevention, the details of the survey design and methodology can be found on the NHANES website [Centers for Disease Control and Prevention (CDC), http://cdc.gov/nchs/nhanes)]. We selected only three cycles of NHANES 2005-2006, 2007-2008, and 2009-2010 to investigate the relationship between perfluorinated alkyl substances concentrations and bone mineral density in adolescents aged 12-19 years, since bone mineral density was measured only for those aged 40 (or 50) years or older from the NHANES 2013-2014 cycle, and bone mineral density of the femur, femoral neck, and lumbar spine was not measured in the 2011-2012 cycle. In the three cycles, a total of 31,034 people participated in the survey, including 4,865 adolescents aged 12-19 years, and only 1,361 had data on serum PFAS, among which we finally selected 1,228 people with complete BMD data on the total femur and femoral neck or lumbar spine (**Figure 1**).

### PFAS measurements

The quantification of PFAS in CDC is derived from a combination of solid-phase extraction and high-performance liquid chromatography-turbine ionization tandem mass spectrometry as also described in other cases (16). Concentrations below the limits of detection (LOD) were replaced with LOD divided by the square root of 2 (17), We selected four PFAS biomarkers that were detected in > 98% of participants: PFOA, PFOS, PFHxS, and PFNA, and we performed a natural logarithmic transformation of the serum PFAS concentrations because they showed a significantly skewed distribution.

### BMD measurements

Dual-energy X-ray absorptiometry (DXA) is the most widely accepted method of measuring bone density due to its speed, ease of use, and low radiation dose (18), and the bone mineral density of the total femur, femoral neck, and lumbar spine is also measured by experienced professional technicians using a dual x-ray absorptiometry technique (QDR 4500A fan-beam densitometers [Hologic Inc]), lumbar bone mineral density is the average of the first to fourth lumbar vertebrae, the detailed measurements for each part can be found on the NHANES website (http://cdc.gov/nchs/nhanes).

### Other covariates

We identified potential confounding factors associated with strong predictors of serum PFAS levels and bone mineral density based on previous studies, which included demographic information such as age, sex, race, and family income to poverty ratio, but also body mass index (BMI), smoking (serum cotinine), exercise status (performing vigorous or moderate exercise), serum lead, albuminuria and anemia, the demographic information was collected from questionnaires administered during home visits. BMI is calculated by dividing body weight (kg) by body height (m^2^), vigorous physical activity was identified from the questionnaire (did you do any vigorous activities for at least 10 minutes that caused heavy sweating, or large increases in breathing or heart rate such as running, lap swimming, aerobics classes or fast bicycling). Moderate physical activity was determined from the questionnaire (did you do moderate activities for at least 10 minutes that cause only light sweating or a slight to moderate increase in breathing or heart rate such as brisk walking, bicycling for pleasure, golf, and dancing).

Continuous variables such as age, income poverty rate, BMI, and serum cotinine were categorized using different criteria when stratifying the covariates, and continuous variable such as serum lead was mainly stratified by quartiles. Families with an income poverty ratio of <1.3 are classified as low-income families, and≥1.3 are classified as middle to high-income families (https://www.cbpp.org/research/food-assistance), for the BMI classification, we used the traditional percentile thresholds from the Centers for Disease Control and Prevention growth charts to identify subjects with a BMI below the 5th percentile as underweight, a BMI between the 5th and 85th percentile as normal weight, a BMI between the 85th and 95th percentile as overweight, and a BMI above the 95th percentile as obese (19), and we classified individuals people with serum cotinine levels <1.0 ng/mL as non-smokers, those with levels between 1.0 and 9.9 ng/mL as people exposed to environmental tobacco smoke (ETS), and those with levels ≥ 10.0 ng/mL as smokers (http://www.cdc.gov/exposurereport). We classified people with an albumin/creatinine ratio (ACR) greater than 30 mg/g as having albuminuria, females with a whole blood hemoglobin concentration <12 g/dL, and males with a whole blood hemoglobin concentration <13 g/dL as having anemia (20, 21).

### Statistical analysis

In all analyses of the article, Continuous variables are represented by the mean standard error (SE), whereas categorical variables are represented by numbers and percentages, and gender differences were tested using the Student’s two-tailed t-test or the Rao–Scott chi-square test. We utilized a multiple regression model to assess the relationship between individual ln-PFAS and the availability of bone mineral density in the total femur, femoral neck, and lumbar spine, results Expressed as regression coefficients and 95% confidence intervals (CI), then divided the ln-PFAS levels into quartiles for quartile-based repeated analyses, and set the lowest quartile as the reference. Since previous studies have shown that the association between PFAS and BMD has been observed mainly in females, we conducted stratified analysis by gender to assess the potential effect modification. Models were adjusted for sex, age, race, income poverty rate, BMI, serum cotinine, vigorous physical activity, moderate physical activity, serum lead, albuminuria, and anemia.

Stratified analyses, as well as a significance test of the interaction term with exposure, were conducted to explore the effect modification by BMI groups [underweight/normal weight (BMI< 85th percentile), overweight/obese (BMI ≥ 85th percentile)], albuminuria (yes/no) and anemia (yes/no), due to these three factors that can affect PFAS and BMD (22–27).

NHANES makes the data collected nationally representative through a complex sampling design and by using sample weights. We weighted the data according to the NHANES recommended sample weight calculation method, the six-year weights for the 2005-2006, 2007-2008, and 2009-2010 estimates were calculated by multiplying the two-year weights by one-third. We used Empowerstats software (www.empowerstats.com) and R (http://www.R-project.org) for all data analysis, the significance of the data is shown by the p-value < 0.05.

## Results

### The research population’s characteristics

All participants were 15.44 ± 2.23 years old on average, non-Hispanic whites make up the majority of the study population (**Table 1**). The average family income poverty rate was 2.71 ± 1.68, with no significant difference in gender. Males exhibited greater rates of smoking and vigorous physical activity than females (all p < 0.05), but females had significantly higher rates of albuminuria and anemia than males (all p <0.05), moderate physical activity and BMI had no significant differences.

**Table 1:** Characteristics of the study population, overall and by sex, NHANES 2005-2010.

Characteristic variable	Overall		Male		Female		p-Value^a^
	n	Mean ± SE or percent	n	Mean ± SE or percent	n	Mean ± SE or percent	
Age (years)	1228	15.44 ± 2.23	670	15.44 ± 2.22	558	15.43 ± 2.25	0.983
Race/ethnicity	1228						0.722
Non-Hispanic white	346	60.04	199	61.74	147	57.84	
Non-Hispanic black	337	13.92	182	13.22	155	14.82	
Mexican American	374	13.33	196	12.6	178	14.28	
Other Hispanic	111	6.08	63	6.02	48	6.14	
Other multiracia	60	6.63	30	6.42	30	6.91	
Income poverty ratio	1147	2.71 ± 1.68	629	2.75 ± 1.65	518	2.67 ± 1.71	0.411
Smoking status^b^	1228		670		558		0.001
Nonsmokers	949	76.35	492	73.24	457	80.38	
ETS	129	9.81	72	9.78	57	9.84	
Smoker	150	13.84	106	16.98	44	9.78	
BMI (kg/m)	1225	23.68 ± 5.75	668	23.78 ± 5.51	557	23.55 ± 6.03	0.501
Serum lead (μg/dL)	1227	0.92 ± 0.71	670	1.07 ± 0.83	557	0.73 ± 0.44	<0.001
Vigorous physical activity	1205		660		545		<0.001
Yes	821	69.42	512	77.64	309	58.71	
No	384	30.58	148	22.36	236	41.29	
Moderate physical activity	1205		660		545		0.173
Yes	683	59.38	375	61.08	308	57.18	
No	522	40.62	285	38.92	237	42.82	
Albuminuria	1223						0.023
Yes	96	6.2	46	4.82	50	7.99	
No	1127	93.8	621	95.18	506	92.01	
Anemia	1221						<0.001
Yes	161	13.88	54	8.92	107	20.27	
No	1060	86.12	610	91.08	450	91.08	
Total femur BMD (g/cm^2^)	1211	0.99 ± 0.16	656	1.02 ± 0.17	555	0.94 ± 0.13	<0.001
Femoral neck BMD (g/cm^2^)	1211	0.91 ± 0.15	656	0.93 ± 0.16	555	0.87 ± 0.13	<0.001
Lumbar spine BMD (g/cm^2^)	1191	0.95 ± 0.15	662	0.93 ± 0.17	529	0.97 ± 0.13	<0.001
PFOA (ng/mL) ^c^	1228	3.80 ± 1.78	670	4.03 ± 1.75	558	3.50 ± 1.79	<0.001
PFOS (ng/mL) ^c^	1228	12.96 ± 9.04	670	14.02 ± 9.65	558	11.60 ± 7.97	<0.001
PFHxS (ng/mL) ^c^	1228	3.88 ± 4.95	670	4.41 ± 5.52	558	3.20 ± 4.01	<0.001
PFNA (ng/mL) ^c^	1228	1.23 ± 0.72	670	1.30 ± 0.77	558	1.14 ± 0.65	<0.001

p-Value^a^, validation of differences between males and females, t-test for continuous variables, and Rao-Scott chi-square test for categorical variables.

Smoking status^b^, classification according to serum cotinine levels.

PFOA (ng/mL)^c^, PFOS (ng/mL)^c^, PFHxS (ng/mL)^c^, PFNA (ng/mL)^c^, untransformed serum perfluoroalkyl concentrations of the environment.

In terms of bone mineral density, the bone mineral density of the total femur and femoral neck in males was 8% and 6% higher than in females respectively, while the bone mineral density of the lumbar spine was 4% lower than in females (p<0.001). Concerning serum PFAS levels, serum PFOA, PFOS, PFHxS, and PFNA levels were 15%, 26%, 37%, and 14% higher in males than in females respectively.

In the **Supplementary Material**, **Table S1** summarizes the different stratified covariates and PFAS concentrations. PFOA was significantly correlated with all stratified covariates except BMI category (all p < 0.05), PFOS was correlated with all stratified covariates except anemia (all p < 0.05), PFHxS was significantly associated with sex, race, family income status, smoking status, vigorous physical activity, albuminuria (all p < 0.05), PFNA was only correlated with sex, race and family income status (all p < 0.05). **Table S2** analyzes the covariates and bone mineral density for the different strata. Total femur and femoral neck BMD were significantly associated with all stratified covariates except family income status, moderate physical activity, serum lead quartiles, and albuminuria (all p< 0.05). In addition to family income status, vigorous physical activity and moderate physical activity lumbar spine BMD were significantly correlated with all stratified covariates (all p< 0.05).

### Associations of PFAS with BMD

**Table 2** shows the outcomes of multivariate regression analysis of ln-transformed serum PFAS with total femur BMD, femoral neck BMD, and lumbar spine BMD separately. In the adjusted model, ln-PFOA, ln-PFOS, and ln-PFNA were negatively correlated with BMD of the three sites (all p< 0.05), and ln-PFHxS was positively correlated with total femur and femoral neck BMD (all p< 0.05). ln-PFOA with lumbar spine BMD, ln-PFOS with BMD of the three sites, and ln-PFNA with lumbar spine BMD after repeated analysis of quartiles in the trend test remained significant (all p for trend < 0.05).

**Table 2:** Association between PFAS concentrations and BMD in young adults aged 12-19 years, NHANES 2005-2010.

ln-PFAS	Total femur BMD	Femur neck BMD	Lumbar spine BMD
n = 1174	β (95% CI)	β (95% CI)	β (95% CI)
ln-PFOA	**-0.017 (-0.031, -0.002)**	**-0.017 (-0.031, -0.003)**	**-0.020 (-0.033, -0.007)**
Q1	Reference	Reference	Reference
Q2	-0.003 (-0.025, 0.020)	0.006 (-0.015, 0.027)	-0.006 (-0.026, 0.014)
Q3	-0.009 (-0.031, 0.013)	-0.006 (-0.028, 0.015)	-0.010 (-0.029, 0.010)
Q4	-0.017 (-0.040, 0.006)	-0.016 (-0.038, 0.006)	-0.026 (-0.046, -0.006)
P for trend	0.095	0.054	**0.008**
ln-PFOS	**-0.021 (-0.032, -0.010)**	**-0.019 (-0.030, -0.009)**	**-0.022 (-0.032, -0.012)**
Q1	Reference	Reference	Reference
Q2	-0.025 (-0.045, -0.004)	-0.021 (-0.041, -0.001)	-0.014 (-0.032, 0.004)
Q3	-0.025 (-0.046, -0.005)	-0.022 (-0.042, -0.002)	-0.017 (-0.036, 0.001)
Q4	-0.051 (-0.072, -0.030)	-0.045 (-0.065, -0.025)	-0.040 (-0.059, -0.022)
P for trend	**<0.001**	**<0.001**	**<0.001**
ln-PFHxS	**0.007 (0.000, 0.014)**	**0.008 (0.001, 0.015)**	-0.004 (-0.010, 0.002)
Q1	Reference	Reference	Reference
Q2	-0.004 (-0.026, 0.017)	-0.011 (-0.032, 0.009)	-0.011 (-0.030, 0.008)
Q3	0.012 (-0.009, 0.034)	0.012 (-0.009, 0.032)	-0.012 (-0.031, 0.007)
Q4	0.017 (-0.005, 0.038)	0.016 (-0.005, 0.037)	-0.015 (-0.034, 0.004)
P for trend	0.058	0.067	0.171
ln-PFNA	**-0.014 (-0.027, -0.001)**	**-0.014 (-0.026, -0.002)**	**-0.016 (-0.027, -0.004)**
Q1	Reference	Reference	Reference
Q2	-0.022 (-0.044, -0.001)	-0.010 (-0.031, 0.011)	-0.021 (-0.040, -0.001)
Q3	-0.014 (-0.036, 0.007)	-0.008 (-0.029, 0.012)	-0.023 (-0.037, 0.001)
Q4	-0.017 (-0.039, 0.004)	-0.013 (-0.034, 0.007)	-0.025 (-0.044, -0.006)
P for trend	0.285	0.268	**0.024**

Adjusted for age, gender, race, income poverty ratio, serum cotinine, vigorous physical activity, moderate physical activity, BMI, serum lead, albuminuria, and anemia.

The bold values indicate significance (p<0.05).

### Subgroup analysis

**Figure 2:**
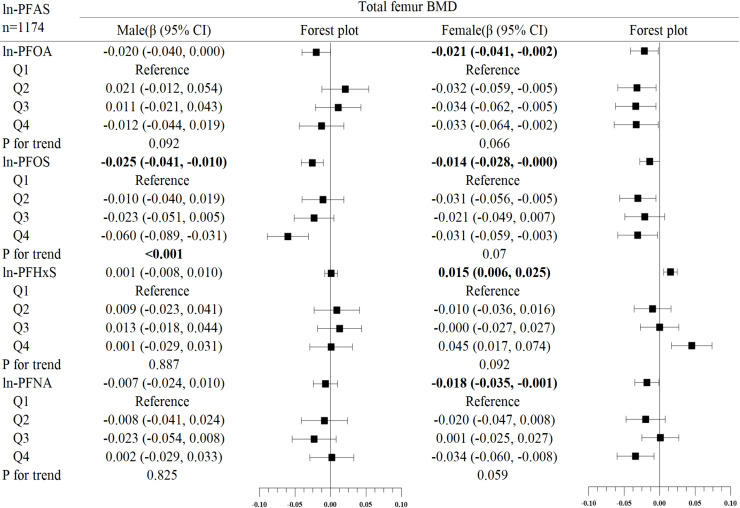
Association between ln-PFOA and bone mineral density, stratified by gender. Adjusted for age, race, income poverty ratio, serum cotinine, vigorous physical activity, moderate physical activity, BMI, serum lead, albuminuria, and anemia. The bold values indicate significance (p<0.05).

**Figure 3:**
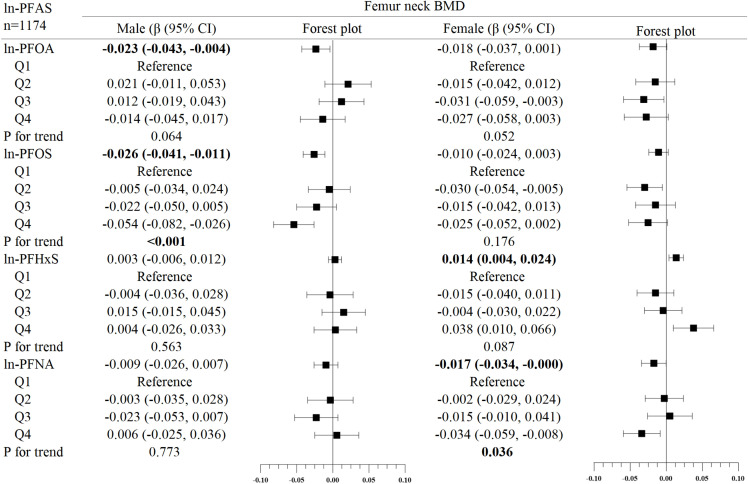
Association between ln-PFOS and bone mineral density, stratified by gender. Adjusted for age, race, income poverty ratio, serum cotinine, vigorous physical activity, moderate physical activity, BMI, serum lead, albuminuria, and anemia. The bold values indicate significance (p<0.05).

**Figure 4:**
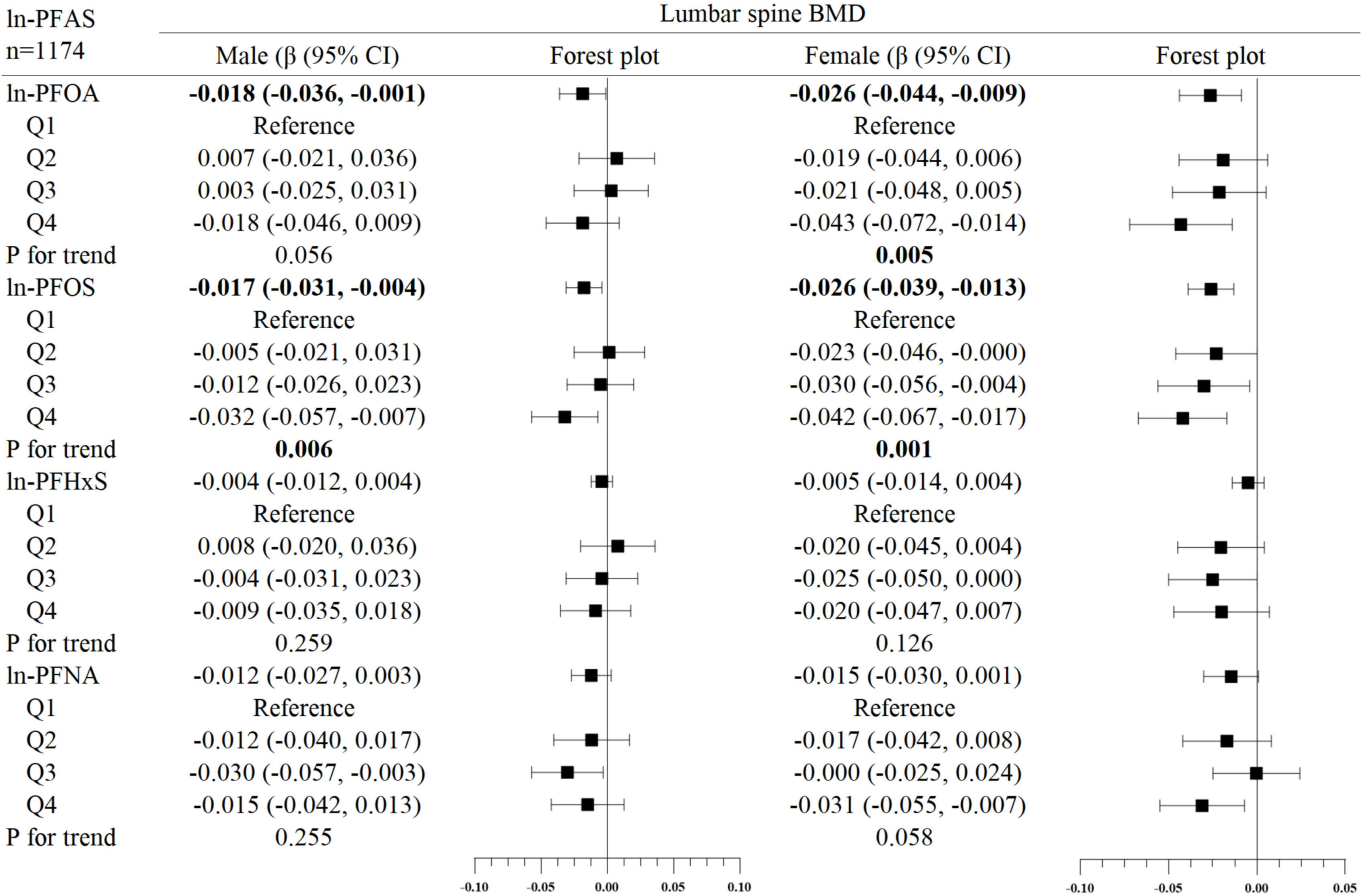
Association between ln-PFHxS and bone mineral density, stratified by gender. Adjusted for age, race, income poverty ratio, serum cotinine, vigorous physical activity, moderate physical activity, BMI, serum lead, albuminuria, and anemia. The bold values indicate significance (p<0.05).

In the subgroup analysis of gender (**Figures 2**–**4**), in males, ln-PFOA was negatively associated with femoral neck and lumbar spine BMD (all p< 0.05), ln-PFOS was associated with reduced total femur, femoral neck, and lumbar spine BMD (all p< 0.05), while ln-PFHxS and ln-PFNA were not observed to correlate with BMD at these three sites. ln-PFOS was significantly associated with the quartile trend of BMD at all three sites in males (all p for trend < 0.05). In females, both ln-PFOA and ln-PFOS were negatively correlated with total femur and lumbar spine BMD (all p< 0.05), ln-PFHxS is associated with the increased total femur and femoral neck BMD (all p< 0.05), and ln-PFNA was negatively correlated with total femur and femoral neck BMD (all p< 0.05). ln-PFOA and ln-PFOS had significant quartile trends with lumbar spine BMD, as well as ln-PFNA with femoral neck BMD (all p for trend < 0.05). By observing the forest plots of PFAS and its quartiles with BMD, we could find that most of the associations were confined to females.

**Table 3** presents the results of the follow-up stratified (BMI, albuminuria, and anemia) analysis, which showed that the associations of ln-PFOS with femoral neck BMD and ln-PFNA with total femur BMD were more significant in those who were overweight/obese and had anemia, respectively (all p for interaction < 0.05), the results were consistent with the results of the regression analysis.

**Table 3:** Association between PFAS concentration and BMD, stratified by BMI groups, albuminuria (yes/no), and anemia (yes/no).

Subgroup	PFOA	PFOS	PFHxS	PFNA
	β (95% CI)	β (95% CI)	β (95% CI)	β (95% CI)
Total femur BMD				
BMI category				
<85th percentile	-0.014 (-0.033, 0.005)	-0.020 (-0.034, -0.006)	0.005 (-0.004, 0.014)	-0.008 (-0.026, 0.010)
≥85th percentile	-0.029 (-0.055, -0.002)	-0.040 (-0.060, -0.020)	0.010 (-0.002, 0.022)	-0.026 (-0.046, -0.006)
P for interaction	0.378	0.111	0.509	0.178
Albuminuria				
Yes	0.007 (-0.028, 0.042)	-0.013 (-0.042, 0.015)	-0.004 (-0.024, 0.016)	0.005 (-0.028, 0.038)
NO	-0.020 (-0.037, -0.004)	-0.021 (-0.033, -0.009)	0.009 (0.002, 0.017)	-0.017 (-0.031, -0.003)
P for interaction	0.157	0.648	0.201	0.226
Anemia				
Yes	-0.035 (-0.076, 0.006)	-0.025 (-0.061, 0.011)	0.002 (-0.026, 0.030)	**-0.072 (-0.127, -0.016)**
No	-0.016 (-0.032, 0.000)	-0.023 (-0.035, -0.011)	0.007 (0.000, 0.015)	-0.011 (-0.024, 0.002)
P for interaction	0.401	0.907	0.721	**0.034**
Femoral neck BMD				
BMI category				
<85th percentile	-0.011 (-0.030, 0.007)	0.017 (-0.030, -0.003)	0.005 (-0.003, 0.014)	-0.006 (-0.023, 0.011)
≥85th percentile	-0.032 (-0.057, -0.006)	**-0.042 (-0.061, -0.022)**	0.012 (0.000, 0.023)	-0.027 (-0.047, -0.007)
P for interaction	0.195	**0.034**	0.407	0.107
Albuminuria				
Yes	0.016 (-0.018, 0.049)	-0.006 (-0.034, 0.021)	-0.005 (-0.024, 0.014)	0.009 (-0.022, 0.041)
NO	-0.024 (-0.040, -0.008)	-0.021 (-0.033, -0.010)	0.010 (0.003, 0.018)	-0.018 (-0.032, -0.005)
P for interaction	0.079	0.332	0.143	0.108
Anemia				
Yes	-0.017 (-0.056, 0.022)	-0.013 (-0.047, 0.022)	0.001 (-0.026, 0.028)	-0.052 (-0.106, 0.001)
No	-0.020 (-0.035, -0.004)	-0.022 (-0.034, -0.011)	0.009 (0.002, 0.016)	-0.012 (-0.025, 0.000)
P for interaction	0.900	0.605	0.576	0.149
Lumbar spine BMD				
BMI category				
<85th percentile	-0.020 (-0.037, -0.003)	-0.023 (-0.035, -0.011)	-0.001 (-0.009, 0.007)	-0.013 (-0.028, 0.003)
≥85th percentile	-0.023 (-0.046, 0.001)	-0.032 (-0.050, -0.014)	-0.008 (-0.018, 0.003)	-0.021 (-0.039, -0.003)
P for interaction	0.834	0.397	0.313	0.489
Albuminuria				
Yes	-0.018 (-0.049, 0.013)	-0.019 (-0.044, 0.007)	-0.021 (-0.039, 0.004)	-0.029 (-0.059, 0.000)
NO	-0.019 (-0.033, -0.004)	-0.021 (-0.031, -0.010)	-0.001 (-0.007, 0.006)	-0.013 (-0.025, -0.000)
P for interaction	0.991	0.877	0.060	0.304
Anemia				
Yes	-0.015 (-0.051, 0.021)	-0.022 (-0.054, 0.010)	-0.001 (-0.026, 0.024)	-0.060 (-0.109, -0.011)
No	-0.021 (-0.035, -0.006)	-0.022 (-0.033, -0.012)	-0.004 (-0.010, 0.003)	-0.013 (-0.024, -0.001)
P for interaction	0.770	0.978	0.831	0.064

Adjusted for age, gender, race, income poverty ratio, serum cotinine, vigorous physical activity, moderate physical activity, BMI, serum lead, albuminuria, and anemia, but not for the stratification variables themselves.

The bold values indicate significance (p<0.05).

## Discussion

In this study, we evaluated the correlation between exposure to specific PFASs (PFOA, PFOS, PFHxS, and PFNA) and bone mineral density in the total femur, femoral neck, and lumbar spine in adolescents aged 12-19 years at NHANES from 2005-2010. Some of the results showed that exposure to PFASs was associated with reduced BMD in adolescents, as indicated by ln-PFOA, ln-PFOS, and ln-PFNA all being negatively associated with total femur, femoral neck, and lumbar spine BMD, which is consistent with our previous hypothesis, the results of the stratified analysis showed that this association was mostly confined to females. Subsequent stratification analysis showed that the associations of ln-PFOS with femoral neck BMD and ln-PFNA with total femur BMD were more significant in overweight/obese, and those with anemia, respectively, while albuminuria conditions did not significantly modulate the association between PFAS and BMD.

Before our study, there were also NHANES studies that reported the association of PFASs exposure with reduced bone mineral density. In a survey of a population aged 8 years and older from 2005 to 2008, higher PFOS serum concentrations were found to be associated with reduced total lumbar spine BMD, primarily in premenopausal women, and no association was detected between serum PFOA, PFOS concentrations with femoral neck BMD (28). In addition, another report from 2009-2010 in a population aged 12-80 years found that serum PFOA, PFOS, PFHxS, and PFNA concentrations were associated with lower total femur and femoral neck BMD in women, while serum PFOA concentrations were associated with lower femoral neck BMD in men, but did not show any clear association between lumbar spine BMD and any PFAS (29).

In addition to the NHANES report, several other epidemiological studies have found an association between exposure to PFASs and reduced bone mineral density, including one study of overweight/obese adolescents aged 8 to 12 years finding that serum PFNA concentrations were significantly and negatively associated with skeletal parameters including broadband-ultrasound attenuation (BUA), the speed of sound waves (SOS), and the stiffness index (SI), which respond to higher bone health and higher BMD (30). Another similar study showed that PFAS exposure was significantly associated with reduced SI in young men (31). Also in a prospective study, PFOA and PFOS were associated with low BMD at several sites including spine, total hip, femoral neck, and hip rotor, and similar correlations were found for PFHxS, PFNA, and perfluorodecanoic acid (PFDA) in the intertransverse region of the hip (32). In addition to reports examining the relationship between a specific population’s autologous PFAS exposure and bone mineral density, several studies have found a negative correlation between serum PFAS concentrations in women exposed prenatally to PFAS and their offspring’s site-specific bone mineral density (14, 33, 34).

Not only epidemiological studies have uncovered the adverse effects of PFASs exposure on bone mineral density, but animal experiments have also reported a similar situation, in which PFOS was able to detectable in bone tissue of adult mice after 1-5 days of dietary exposure, and in addition, pregnant rats and mice exposed prenatally to PFOS showed fetal skeletal malformations as well as a decrease in bone mineral density (35–37), in addition, the results of human tissue examinations are similar to the findings of population studies, which have shown that PFASs can deposit in bone tissue and accumulate over time to exhibit some toxic effects, thereby affecting bone health (38–40).

The potential mechanisms of PFAS for adverse skeletal effects are not yet clear (11), and current studies have confirmed possible mechanisms that encompass several aspects, the first of which is the direct effect of PFAS on bone, current *in vivo* and *in vitro* studies on humans and animals have demonstrated that PFOA can take direct action on bone and bone marrow cells. In animal studies, for osteoblasts, the effects of different concentrations of PFOA on osteocalcin (OCN) expression and calcium secretion were dramatically different, as indicated by promotion at low concentrations and inhibition at high concentrations. In contrast, for osteoclasts, their number increased at all PFOA concentrations tested, but their resorption activity increased at low PFOA concentrations, decreased, and finally stopped at high concentrations (37). In terms of the effect of PFOA on osteoblasts, the results of human *in vitro* experiments were consistent with those of animal experiments, but PFOA did not interfere with osteogenic differentiation (40). PFOA also impairs the differentiation of hematopoietic stem cells and the stereotyping of bone marrow mesenchymal stem cells (41). Although very few studies have been conducted on osteoclast and osteoblast changes associated with PFAS exposure, PFOS, PFHxS, and PFOA have also been reported to affect multiple pathway targets (mRNA and protein of RUNX2), thereby inhibiting osteoblast differentiation (42). In addition, low concentrations of PFOS can achieve the same effect by decreasing the expression of the mRNAs for the osteoblast biomarkers bone bridging protein and bone junction protein (43), and PFOA and can also affect osteoblast function by significantly reducing alkaline phosphatase activity, collagen synthesis and mineralization in osteoblasts (44).

The second is that PFAS affects the skeleton through endocrine disruptive effects, mainly in both sex hormones and thyroid hormones (11), as both of them can significantly affect bone remodeling and bone health (45, 46). Both laboratory and epidemiological studies have now found a strong correlation between PFAS and sex hormones (47–53), for example, PFAS has a strong correlation with delayed puberty, early menopause, and serum estradiol concentration (54, 55), this may also explain the difference in the association between PFAS and BMD between the sexes in our study, and a large number of studies have confirmed that PFAS can also have significant effects on thyroid hormones (56–58), such as the association of PFAS with thyroxine (T4) and triiodothyronine (T3) levels (56).

Besides, recent *in vitro* evidence suggests that PFOA can interfere with the action of vitamin D by binding directly to hydroxyapatite crystals (59), along with epidemiological studies reporting that PFAS is associated with lower levels of total 25-hydroxyvitamin D (60), the latter is closely associated with bone health (61), so this could be another potential mechanism by which PFAS affects bone. It has also been shown that PFAS can impair osteoblast formation by activating peroxisome proliferator-activated receptor-γ (PPARγ), thereby affecting bone health (62).

Another important finding of our study, in addition to reporting the above association, is that PFAS exposure was associated with higher BMD in adolescents, specifically, serum PFHxS was associated with higher BMD in the total femur and femoral neck, which seems to contradict our previous hypothesis. Although fewer studies have been reported to demonstrate that PFAS exposure is positively associated with BMD, there is still an NHANES study similar to our results, and their finding showed that PFOA, PFOS, PFHxS, and PFDE were negatively associated with proximal femoral BMD in premenopausal women, whereas PFOA, PFOS, PFHxS, and PFNA were positively associated with proximal femoral BMD in men (63). There are very few studies on the mechanisms underlying the positive effects of PFASs exposure on bone health. However, there is still experimental demonstration that some PFAS at low concentrations is associated with increased OCN expression and calcium secretion, which facilitates osteogenesis (37), and there are also studies showing that some PFAS are associated with increased FT4 (64), which may inhibit TSH, a decrease in which may contribute to osteoporosis. Therefore, the above speculations may explain this association.

Another interesting finding is that the association between PFAS and BMD is strengthened in those who are overweight/obese and have anemia, but not in those who have albuminuria the mechanisms of how PFAS and obesity interact remain unclear, but obesity can affect bone health through multiple pathways, and there may be a synergistic effect with PFAS in one of these pathways to affect bone health, such as hormone secretion (65, 66), and altered tissue distribution of PFAS in more obese populations may also influence its effect on bone health (67), all of these may help explain the enhanced effect of PFAS on BMD in overweight/obese populations. There are few studies on the interaction between albuminuria and PFAS, but some studies have shown that albuminuria is associated with reduced bone blood flow, which leads to a reduced rate of bone remodeling and the development of osteoporosis (68), and also that renal failure with albuminuria may lead to less renal reabsorption, which may have an impact on PFAS excretion, thus affecting serum PFAS levels (69). However, our study did not find that the association between PFAS and BMD was strengthened in the population with albuminuria. It has been suggested that anemia may affect serum PFAS levels (70), and have an effect on BMD (27), our study confirms such findings, but the exact mechanism is not explained by current studies, so more studies are needed to confirm these findings.

Some differences can be observed by comparing our study with previous NHANES reports. Our study found that in males, PFOA was negatively associated with femoral neck and lumbar spine BMD, PFOS was negatively associated with total femur, femoral neck, and lumbar spine BMD, PFHxS and PFNA were not associated with BMD at any of these three locations. In females, both PFOA and PFOS were associated with the reduced total femur and lumbar spine BMD, PFHxS and PFNA were positively and negatively associated with total femur and femoral neck BMD, respectively, but Lin’s study reported that PFOS was not associated with femoral neck BMD (28), Khalil’s study demonstrated that PFOA was associated with the reduced total femur and femoral neck BMD and not lumbar spine BMD in females, PFOS was also not associated with lumbar spine BMD, and PFHxS and PFNA were associated with the reduced total femur and femoral neck BMD in females (29). The above differences may be due to variations in NHANES survey period, survey sample size, age group, and different covariates.

Our study has some strengths, first, as far as we know, the correlation of autologous PFAS levels with bone mineral density in adolescents has never been explored separately, and this is the first study to do so. Second, we explored the role of the effect of different populations on the association between PFAS and BMD by stratifying the data for multiple comparisons. Third, we quantified the independent variables and performed trend tests, and also performed interaction tests after stratified analysis, which reduced the chances of data analysis and enhanced the robustness of the results.

However, our current analysis has some limitations. First, due to the cross-sectional nature of the study. We were unable to identify the causal relationship between serum PFOS levels and BMD. Second, in our analysis of subsequent stratification (BMI, proteinuria, and anemia), we did not fail to replicate the analysis for gender differences to derive differences in the effects of obesity, proteinuria, and anemia on PFAS and BMD by gender. Third, although potential confounding factors are considered, we cannot completely exclude residual and unmeasured confounding factors. Fourth, some of our covariate data, in spite of being collected by trained interviewers with standardized protocols, are still subject to self-report bias.

## Conclusions

In conclusion, PFAO, PFOS, and PFNA were associated with lower BMD and PFAS with higher BMD in US adolescents aged 12-19 years, and these associations were mostly confined to females, and the negative effect of PFAS on BMD was more pronounced in those who were overweight/obese and had anemia however, additional laboratory and prospective epidemiological studies are needed to confirm these findings.

## Data availability statement

The original contributions presented in the study are included in the article/**Supplementary Material**. Further inquiries can be directed to the corresponding author.

## Ethics statement

The studies involving human participants were reviewed and approved by NHANES. The patients/participants provided their written informed consent to participate in this study.

## Author contributions

This study was designed by YG. XX extracted the associated data from NHANES. BC performed the statistical analysis. XX completed the composition of the manuscript, helped supervised the analysis, and revised and approved the manuscript. All authors contributed to the article and approved the submitted version.

## Funding

This study was supported by the Natural Science Foundation of Guangdong Province (2018A030313369). 2022 Huang Feng National Famous Traditional Chinese Medicine Expert Inheritance Studio. Special Funds for the Discipline Reserve Talent Cultivation Project of Guangzhou University of Chinese Medicine “Double First-Class” and High-level University Construction; National Natural Science Foundation of China (81974575).

## Acknowledgments

We thank the NHANES data for being publicly available on the Internet for use by researchers worldwide.

## Conflict of interest

The authors declare that the research was conducted in the absence of any commercial or financial relationships that could be construed as a potential conflict of interest.

## Publisher’s note

All claims expressed in this article are solely those of the authors and do not necessarily represent those of their affiliated organizations, or those of the publisher, the editors and the reviewers. Any product that may be evaluated in this article, or claim that may be made by its manufacturer, is not guaranteed or endorsed by the publisher.

## Abbreviations

NHANES, National Health and Nutrition Examination Survey; PFOA, Perfluorooctanoic acid; PFOS, Perfluorooctane sulfonic acid; PFHxS, Perfluorohexane sulfonic acid; PFNA, Perfluorononanoic acid; BMD, Bone mineral density; PFASs, Perfluoroalkyl substances; Ln, Natural log; EDCs, Endocrine-disrupting chemicals; CDC, Centers for Disease Control and Prevention; LOD, Limit of detection; DXA, Dual-energy X-ray absorptiometry; BMI, Body mass index; ACR, Albumin/creatinine ratio; ETS, Environmental tobacco smoke; SE, Standard error; β, Regression coefficients; OR, Odds ratio; CI, Confidence intervals; BUA, Broadband-ultrasound attenuation; SOS, Speed of sound waves; SI, Stiffness index; PFDA, Perfluorodecanoic acid; OCN, Osteocalcin; PPARγ, Peroxisome proliferator-activated receptor-γ; PFDE, Perfluorodecanoic acid.
