# Supplementary material for: Association between perfluoroalkyl substances concentration and bone mineral density in the US adolescents aged 12-19 years in NHANES 2005-2010
Source: Front Endocrinol (Lausanne). 2022 Oct 5;13:980608. doi: 10.3389/fendo.2022.980608 (PMC9581310; doi:10.3389/fendo.2022.980608)
Supplement: Supplementary file 1 [file DataSheet_1.pdf]

Supplemental Material

Association between perfluoroalkyl substances concentration and bone mineral density in the US adolescents aged 12-19 years in NHANES 2005-2010

Xianmei Xiong[1], Baihang Chen[1], Zhongqing Wang[1], Liqiong Ma[1], Shijie Li[1], Yijia Gao[2]*

Table S1. Mean concentrations of serum perfluoroalkyl substances in different stratified populations (± SE).

Table S2. Mean bone mineral density in different strata of the population (± SE)

Table S1. Mean concentrations of serum perfluoroalkyl substances in different stratified populations (± SE).

Characteristic	PFOA	PFOS	PFHxS	PFNA
Sex				
Male	4.01 ± 1.91	13.67 ± 9.60	3.78 ± 5.27	1.25 ± 0.76
Female	3.20 ± 1.65	11.11 ± 7.70	2.78 ± 3.59	1.05 ± 0.63
P-value	**< 0.001**	**< 0.001**	**< 0.001**	**< 0.001**
Age(years)				
12-15	3.47 ± 1.70	11.68 ± 7.88	3.29 ± 5.08	1.16 ± 0.72
16-19	3.81 ± 1.97	13.34 ± 9.72	3.36 ± 4.09	1.16 ± 0.69
P-value	**0.001**	**0.001**	0.802	0.961
Race/ethnicity				
Non-Hispanic white	4.09 ± 1.85	14.72 ± 10.18	4.61 ± 5.18	1.23 ± 0.75
Non-Hispanic black	3.65 ± 1.92	14.05 ± 8.91	3.17 ± 3.95	1.17 ± 0.63
Mexican American	3.34 ± 1.82	10.39 ± 7.57	2.83 ± 5.06	1.04 ± 0.63
Other Hispanic	3.41 ± 1.48	8.67 ± 6.24	1.88 ± 2.21	1.27 ± 0.82
Other multiracia	3.26 ± 1.65	11.26 ± 6.79	2.50 ± 3.10	1.23 ± 0.96
P-value	**< 0.001**	**< 0.001**	**< 0.001**	**< 0.001**
Family income status				
Low income status	3.30 ± 1.55	10.94 ± 7.41	2.87 ± 3.95	1.11 ± 0.68
Mid-high income status	3.89 ± 1.97	13.80 ± 9.74	3.75 ± 5.16	1.20 ± 0.75
P-value	**< 0.001**	**< 0.001**	**0.002**	**0.025**
BMI category				
Underweight	3.96 ± 1.95	15.11 ± 9.46	3.31 ± 4.74	1.29 ± 0.88
Normal weight	3.73 ± 1.85	13.36 ± 9.45	3.46 ± 4.81	1.15 ± 0.61
Overweight	3.40 ± 1.77	10.95 ± 7.32	2.96 ± 4.11	1.12 ± 0.73
Obese	3.57 ± 1.85	11.36 ± 8.14	3.27 ± 4.49	1.19 ± 0.88
P-value	0.098	**< 0.001**	0.590	0.533
Smoking status				
Nonsmokers	3.53 ± 1.75	12.21 ± 8.82	3.17 ± 4.60	1.14 ± 0.70
ETS	3.54 ± 1.85	11.55 ± 6.53	3.18 ± 3.62	1.15 ± 0.71
Smoker	4.44 ± 2.18	15.17 ± 10.41	4.41 ± 5.30	1.27 ± 0.73
P-value	**< 0.001**	**< 0.001**	**0.008**	0.103
Vigorous physical activity				
Yes	3.78 ± 1.86	13.19 ± 8.87	3.56 ± 5.01	1.18 ± 0.71
No	3.36 ± 1.79	11.23 ± 8.93	2.92 ± 3.71	1.12 ± 0.70
P-value	**< 0.001**	**< 0.001**	**0.026**	0.146
Moderate physical activity				
Yes	3.78 ± 1.92	13.26 ± 8.97	3.55 ± 4.76	1.16 ± 0.76
No	3.47 ± 1.73	11.66 ± 8.81	3.10 ± 4.49	1.16 ± 0.63
P-value	**0.003**	**0.002**	0.097	0.824
Serum lead (μg/dL) quartiles				
=<0.18	3.31 ± 1.66	11.41 ± 8.13	3.63 ± 5.57	1.08 ± 0.66
0.18 =<0.55	3.70 ± 1.91	12.88 ± 8.93	3.24 ± 4.07	1.17 ± 0.68

0.55 =<0.77	3.77 ± 1.73	13.79 ± 10.19	3.53 ± 4.97	1.23 ± 0.74
>0.77	3.77 ± 2.01	11.90 ± 7.90	2.92 ± 3.66	1.15 ± 0.73
P-value	**0.006**	**0.005**	0.221	0.060
Albuminuria				
Yes	3.3 ± 1.7	10.9 ± 6.6	2.6 ± 2.7	1.1 ± 0.8
No	3.7 ± 1.8	12.7 ± 9.1	3.4 ± 4.8	1.2 ± 0.7
P-value	**0.017**	**0.017**	**0.043**	0.270
Anemia				
Yes	3.0 ± 1.7	11.2 ± 9.6	3.0 ± 5.1	1.1 ± 0.6
No	3.7 ± 1.8	12.6 ± 8.8	3.4 ± 4.6	1.2 ± 0.7
P-value	**0.001**	0.140	0.413	0.204

The bold indicates significance (P < 0.05).

PFOA, PFOS, PFHxS, PFNA, untransformed serum perfluoroalkyl concentrations of the environment.

Table S2. Mean bone mineral density in different strata of the population (± SE).

Characteristic	Total femur BMD	Femur neck BMD	Lumbar spine BMD
Sex			
Male	1.03 ± 0.18	0.94 ± 0.17	0.93 ± 0.17
Female	0.95 ± 0.14	0.88 ± 0.14	0.98 ± 0.13
P-value	**< 0.001**	**< 0.001**	**< 0.001**
Age(years)			
12-15	0.93 ± 0.15	0.86 ± 0.14	0.87 ± 0.15
16-19	1.05 ± 0.16	0.97 ± 0.16	1.04 ± 0.12
P-value	**< 0.001**	**< 0.001**	**< 0.001**
Race/ethnicity			
Non-Hispanic white	0.98 ± 0.15	0.90 ± 0.14	0.95 ± 0.15
Non-Hispanic black	1.05 ± 0.18	0.96 ± 0.17	0.99 ± 0.16
Mexican American	0.97 ± 0.16	0.90 ± 0.15	0.93 ± 0.15
Other Hispanic	0.97 ± 0.14	0.89 ± 0.14	0.92 ± 0.16
Other multiracia	0.95 ± 0.18	0.87 ± 0.17	0.93 ± 0.18
P-value	**< 0.001**	**< 0.001**	**< 0.001**
Family income status			
Low income status	0.98 ± 0.16	0.91 ± 0.15	0.95 ± 0.16
Mid-high income status	1.00 ± 0.17	0.92 ± 0.16	0.95 ± 0.16
P-value	0.253	0.383	0.598
BMI (kg/m) category			
Underweight	0.79 ± 0.12	0.72 ± 0.11	0.78 ± 0.14
Normal weight	0.96 ± 0.16	0.88 ± 0.15	0.93 ± 0.15
Overweight	1.02 ± 0.16	0.94 ± 0.15	0.98 ± 0.16
Obese	1.06 ± 0.15	1.00 ± 0.15	1.01 ± 0.15
P-value	**< 0.001**	**< 0.001**	**< 0.001**
Smoking status			
Nonsmokers	0.98 ± 0.16	0.90 ± 0.15	0.94 ± 0.16
ETS	1.02 ± 0.17	0.95 ± 0.16	0.97 ± 0.16
Smoker	1.06 ± 0.17	0.98 ± 0.16	1.03 ± 0.14
P-value	**< 0.001**	**< 0.001**	**< 0.001**
Vigorous physical activity			
Yes	1.01 ± 0.17	0.92 ± 0.16	0.95 ± 0.16
No	0.96 ± 0.14	0.90 ± 0.14	0.96 ± 0.15
P-value	**< 0.001**	**0.003**	0.186
Moderate physical activity			
Yes	1.00 ± 0.17	0.92 ± 0.16	0.95 ± 0.16
No	0.99 ± 0.16	0.91 ± 0.16	0.96 ± 0.16
P-value	0.685	0.685	0.558
Serum lead (μg/dL) quartiles			
=<0.18	0.98 ± 0.16	0.90 ± 0.15	0.97 ± 0.14
0.18 =<0.55	1.01 ± 0.16	0.94 ± 0.15	0.98 ± 0.14

0.55 =<0.77	0.99 ± 0.18	0.91 ± 0.16	0.93 ± 0.16
>0.77	0.98 ± 0.17	0.91 ± 0.16	0.92 ± 0.18
P-value	0.143	0.054	**< 0.001**
Albuminuria			
Yes	0.94 ± 0.15	0.87 ± 0.16	0.92 ± 0.15
No	1.00 ± 0.17	0.92 ± 0.16	0.96 ± 0.16
P-value	**0.001**	**0.001**	**0.004**
Anemia			
Yes	0.97 ± 0.16	0.90 ± 0.15	0.91 ± 0.19
No	0.99 ± 0.17	0.92 ± 0.16	0.96 ± 0.15
P-value	0.111	0.313	**0.006**

The bold indicates significance (P < 0.05).
